# Spike-Timing Dependent Plasticity in Unipolar Silicon Oxide RRAM Devices

**DOI:** 10.3389/fnins.2018.00057

**Published:** 2018-02-08

**Authors:** Konstantin Zarudnyi, Adnan Mehonic, Luca Montesi, Mark Buckwell, Stephen Hudziak, Anthony J. Kenyon

**Affiliations:** Department of Electronic and Electrical Engineering, University College London, London, United Kingdom

**Keywords:** resistive switching, resistance switching, STDP, RRAM, machine learning, neuromorphic systems

## Abstract

Resistance switching, or Resistive RAM (RRAM) devices show considerable potential for application in hardware spiking neural networks (neuro-inspired computing) by mimicking some of the behavior of biological synapses, and hence enabling non-von Neumann computer architectures. Spike-timing dependent plasticity (STDP) is one such behavior, and one example of several classes of plasticity that are being examined with the aim of finding suitable algorithms for application in many computing tasks such as coincidence detection, classification and image recognition. In previous work we have demonstrated that the neuromorphic capabilities of silicon-rich silicon oxide (SiO_x_) resistance switching devices extend beyond plasticity to include thresholding, spiking, and integration. We previously demonstrated such behaviors in devices operated in the unipolar mode, opening up the question of whether we could add plasticity to the list of features exhibited by our devices. Here we demonstrate clear STDP in unipolar devices. Significantly, we show that the response of our devices is broadly similar to that of biological synapses. This work further reinforces the potential of simple two-terminal RRAM devices to mimic neuronal functionality in hardware spiking neural networks.

## Introduction

Non-von Neumann computing architecture, inspired by the neuronal architecture of the brain, is receiving considerable interest as a more efficient way to tackle a range of machine learning tasks such as classification, sorting, and image recognition (Indiveri et al., 2006; Smith, 2006; Izhikevich and Edelman, 2008; Ananthanarayanan et al., 2009; Jo et al., 2010). Such work is not limited to purely software implementations: hardware “neuromorphic” systems, which mimic specific functionalities of biological neurons, most often synaptic plasticity, have been increasingly studied in recent years (Mead, 1990; Indiveri, 2000; Le Masson et al., 2002; Vogelstein et al., 2008; Mitra et al., 2009; Indiveri et al., 2011).

The biological synapse can be naively modeled as a two-terminal structure that permits communication between two neurons via electrical or chemical signals (Chua, 1971; Strukov et al., 2008; Schacter et al., 2011) and whose connection strength depends on electrical history, encoded in the dynamics of ion diffusion. Likewise, Resistive Random Access Memory devices (Waser and Aono, 2007), often described as memristors (Chua, 1971), which are a promising novel non-volatile memory technology (Torrezan et al., 2011; Chen et al., 2012; Mehonic et al., 2012a), are also two-terminal devices whose conductance can be modified dynamically by changes in both ion and electron current flow.

Spike timing dependent plasticity is a form of Hebbian learning rule that results in synaptic connection strength modification based on the relative timing of voltage spikes between neurons (Hebb, 1949; Bi and Poo, 1998; Abbott et al., 2000), more details of which are given below. In recent years, there have been multiple reports of RRAM devices showing STDP behavior in a range of bipolar devices and materials (Jo et al., 2010; Yu et al., 2011, 2013; Ambrogio et al., 2013; Indiveri et al., 2013; Saïghi et al., 2015; Serb et al., 2016). In most studies, memristive devices are operated purely in bipolar mode, in which potentiation and depression of synaptic strength are governed by positive and negative voltage pulses, respectively. However, in order to implement other neuronal functions, including thresholding, spiking and integration, more complex CMOS circuitry is required in such systems to interface with the RRAM “synapse.” Nevertheless, in our previous work we have demonstrated how this broader class of functions can be realized in unipolar SiO_x_ RRAM devices (Mehonic et al., 2015). In the majority of other reported examples of the use of RRAM devices in neuromorphic systems, the role of the RRAM element is to model the behavior of the biological synapse—that is, to provide a programmable synaptic weight in the form of an adjustable conductance. As is the case with the biological system, this has most conveniently been achieved by operating in the bipolar mode. However, oxide-based RRAM devices exhibit interesting and useful behavior when operated in a unipolar mode. In the case of SiO_x_, devices biased with a constant current can enter a metastable state in which field-driven filament formation competes with current-driven filament (partial) dissolution. Adjusting the bias correctly enables us to generate voltage spikes with a current-controlled frequency, removing the need for much of the CMOS electronics used in conventional systems to model neuronal function. While much work remains to be done in this field, the reduction of CMOS circuitry footprint in neuromorphic systems could yield savings in valuable silicon chip real-estate. Other benefits such as power reduction are more uncertain, and remain so until further work has been performed on algorithm development and a fuller understanding of how devices can be designed to operate at very low current and/or duty cycle. Nevertheless, a reduction in circuit complexity by exploiting simple two-terminal devices is an attractive goal. Building on our previous work, here we report, for the first time, spike timing dependent plasticity in such unipolar devices—a result that, when combined with neuronal functionality, promises a greatly reduced component count for RRAM hardware spiking neural networks. Further, we present a statistical analysis of STDP conductance modification mimicking the response of a biological synapse. RRAM devices in our experiments are based on SiO_x_ switching layers; this ensures full compatibility with CMOS technology (Mehonic et al., 2012a). This study serves as a proof of principle of device functionality; energy efficiency will be studied in our future work.

## Materials and methods

All experiments were carried out on metal/SiO_x_/metal devices whose structure is shown schematically in **Figure 2A**. One hundred nanometers of thick titanium nitride (TiN) electrodes sandwich a 37 nm thick SiO_x_ resistance switching layer. All layers were sputter deposited (the SiO_x_ layer was co-sputtered from Si and SiO_2_ targets). The SiO_x_ active switching layer was amorphous and sub-stoichiometric (x ≈ 1.3). The top TiN layer was patterned using conventional lithography techniques into square electrode structures with edges ranging from 100 to 400 μm (for more details of fabrication see Mehonic et al., 2015; Montesi et al., 2016).

Devices were operated in ambient conditions, exhibiting intrinsic switching through the formation of oxygen vacancy filaments, and may therefore be classed as valence change memory (VCM) systems (Mehonic and Kenyon, 2015). Pristine devices require an electroforming step to generate a nanometer-scale conductive oxygen vacancy filament, after which devices may be cycled many times between multiple resistance states (Waser, 2012). During electroforming, current was limited to avoid destructive breakdown of the oxide. Following electroforming, the device enters a low resistance state (LRS). To make a transition from the LRS to a high resistance state (HRS) a lower bias voltage, with no current compliance, is applied to re-oxidize a section of the conductive filament by increasing oxygen ion mobility via Joule heating. To reinstate the filament connection between the anode and cathode a localized process similar to electroforming can be driven by the application of an appropriate field, limited to avoid currents sufficiently high to damage the oxide through catastrophic dielectric breakdown. Further details of the resistance switching mechanism are found in Mehonic et al. (2012a), and a study of the role of oxygen movement in electroforming and switching is in Mehonic et al. (2016).

While characterization of RRAM devices is often carried out using voltage sweeps, in normal operation devices are programmed using short voltage pulses, which may be of the order of nanoseconds in duration. In this case, the major control factors for pulsed electroforming are duration and voltage amplitude of the pulse. We used a Keithley 4200-SCS Semiconductor Characterization System with a Pulse Measure Unit (PMU) to create custom pulse shapes. The PMU does not allow manual control of current compliance, which we implemented instead by carefully controlling pulse voltage and duration. We used custom Matlab scripts to control the system either directly or through custom C drivers. In all cases we contacted devices with tungsten needle probes on a Signatone probe station. Devices were tested in ambient conditions in an isolated low-light environment.

## Results and discussion

### Unipolar resistance switching

Details of resistance switching in intrinsic oxide RRAM devices may be found elsewhere (Mehonic et al., 2012a,b, 2015, 2016, 2017) but to briefly summarize, application of an initial electroforming field, limited to prevent catastrophic dielectric breakdown, across a pristine, highly insulating, oxide generates a filament of conductive oxygen vacancies bridging the oxide film. Subsequent application of a field, either of the opposite polarity (bipolar operation) or similar polarity (unipolar) without a current limit resets the device to a high resistance state. Applying a field of the original polarity (bipolar operation) or the same polarity with a current limit (unipolar) sets the device back to the low resistance state. Devices may typically be cycled between the high and low resistance states many times.

**Figure 1:**
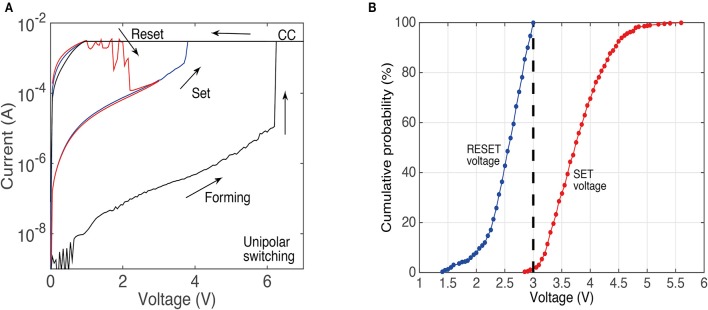
**(A)** Typical I/V curve for devices used in this study showing resistance switching in the unipolar mode. The current compliance limit is labeled as CC. **(B)** Cumulative probability plots of set and reset voltages obtained from more than 1,000 switching cycles (during the I/V sweeps).

Figure 1A shows a typical unipolar resistance switching of the devices we use in this study. The electroforming process typically occurs around 6 V, while the set and the reset voltages are typically 4 and 2.5 V, respectively. For the electroforming and the set process, a current limit of 3 mA is used to prevent hard breakdown. Similar to other RRAM devices, SiO_x_ unipolar devices show a variability of the switching voltages. This is shown in the cumulative plots in Figure 1B, in which data is obtained from more than 1,000 I/V sweeps (top contacts size 200 × 200 μm).

### Setting and resetting dependence on pulse shape

**Figure 2:**
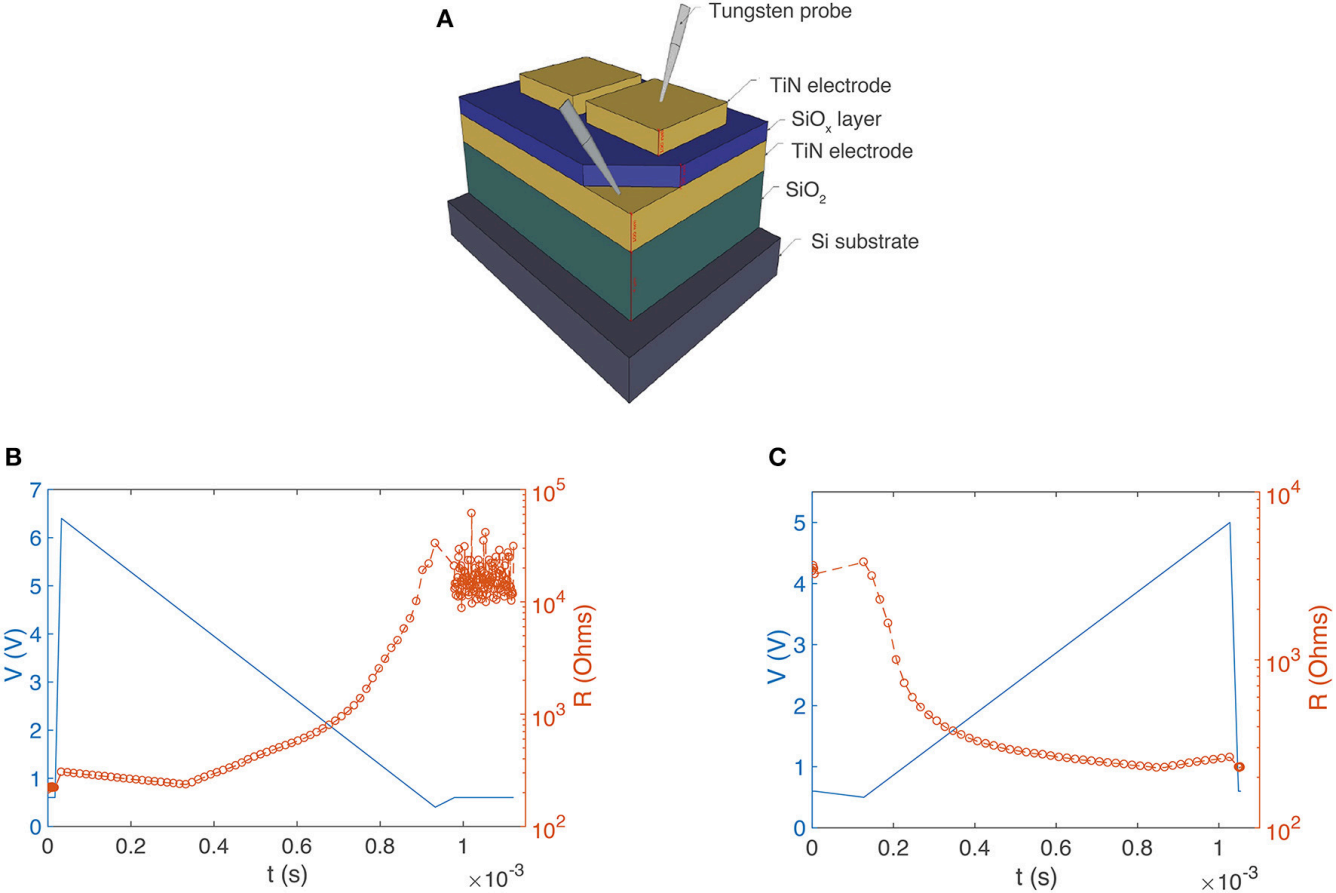
**(A)** Schematic illustration of a SiO_x_ RRAM device (not to scale). TiN layers (electrodes) are 100 nm thick (yellow). The active SiO_x_ layer is ~37 nm thick (blue). The gray needles represent tungsten probes. Controlling device resistance by SET and RESET with leading and trailing edges of voltage pulses. **(B)** Descending train of 300 ns voltage pulses (6.4–0 V: blue line for clarity) increases device resistance from ~100 Ω to ~20 kΩ (orange data points). **(C)** Ascending train of 300 ns voltage pulses (0–5 V: blue line for clarity) decreases device resistance from ~4 k to ~200 Ω (orange data points).

Devices may be set or reset depending on the shape of voltage pulses. Devices may be set by an ascending sequence of 300 ns voltage pulses, which, over a sufficiently long time, look like the leading edge of a voltage pulse, and then they may be reset in response to a descending sequence of pulses that look like the trailing edge of a triangular or sawtooth pulse. The gradual resistance change can be tracked by applying short read voltage pulses (0.6 V, 300 ns) that do not induce any switching (shown in in **Figure 4**). These pulses were applied in between each of the ascending or descending pulses that make up the sawtooth voltage ramp (Figures 2B,C—shown as a continuous blue line for clarity) while the current supplied by the Keithley system was measured, and hence the device resistance obtained. The transition from LRS to HRS begins to occur at around 2.7 V, a voltage that agrees well with the statistical measurements we carried out shown in Figure 2B. The ascending pulse train can then set the device to a LRS (Figure 2C). Resistance gradually decreases as the voltage bias increases, which demonstrates the system's capacity to exhibit plasticity. An important feature is that the resistance value for any SiO_x_ device can be incrementally adjusted by controlling the initial resistance state, voltage starting position and cut-off voltage of the pulse train.

### Spike-timing dependent plasticity (STDP)

That the sign of voltage pulse gradient control both setting and resetting processes in our devices suggests that combining suitable tailored pulses and applying these to individual devices may allow us to emulate STDP in biological synapses. In this study, external circuitry is used to provide programming voltage pulses, but it should be borne in mind that they may in principle be generated by optimized unipolar devices, as we have previously demonstrated (Mehonic and Kenyon, 2016).

STDP is a form of Hebbian learning rule that results in synaptic modification based on the relative timing of pre- and post-synaptic voltage spikes (Hebb, 1949; Bi and Poo, 1998; Abbott et al., 2000; Jo et al., 2010). That is to say, the relative arrival time of two spikes (“pre-synaptic” and “post-synaptic”) at the synaptic gap determines whether the synaptic strength will be increased, decreased, or remain unaltered. The change of state in a biological synapse is represented the change in excitatory postsynaptic current amplitude; in a RRAM device this change is represented by the change in device conductance (Chua, 1971).

**Figure 3:**
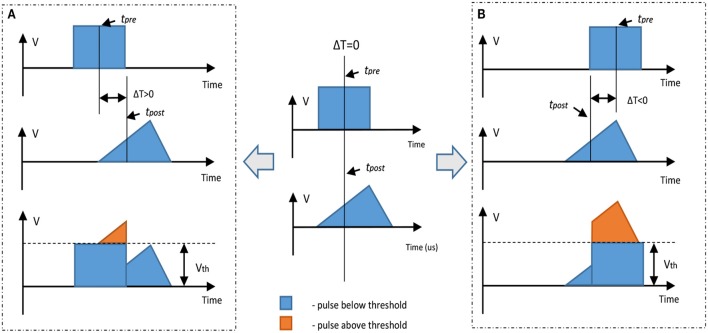
Illustration of a non-identical STDP-mimicking pulse set up. If a square pulse and a triangular pulse are below threshold there is no change in device resistance if a single pulse is applied; however, if the sum of these two pulses is above the threshold it is possible to adjust device resistance. In these examples the square pulse is a pre-synaptic spike and triangle pulse is a post-synaptic spike. **(A)** Pre-synaptic spike arrives earlier than the post-synaptic spike; the resulting sum is a slow leading edge above the threshold. This leads to decrease in resistance (increase in conductance—SET process). **(B)** The post-synaptic spike arrives earlier than the pre-synaptic spike; the resulting sum is a slow trailing edge above the threshold. This leads to an increase in resistance (decrease in conductance—RESET process).

**Figure 4:**
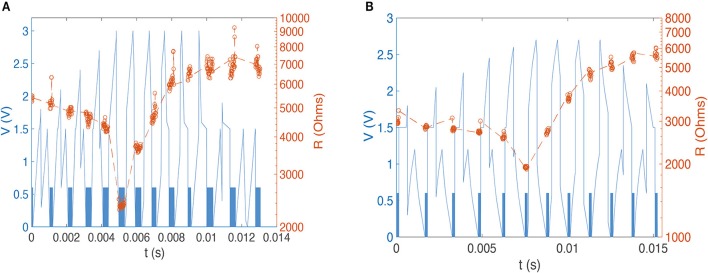
**(A)** Experimental device resistance measurements for a sequence of 12 programming pulses with varying pre-post (square-triangle) delays. Square 0.6 V 300 ns reading pulses (indistinguishable on this time scale, so present as a blue band) between shaped programming pulses yield 450 resistance measurements. We see clear evidence of potentiation and depression depending on programming pulse shape. **(B)** Experimental resistance measurement for a sequence of 11 pulses with varying pre-post delays in which the post-synaptic spike is altered with an emulated capacitor. We see again evidence of potentiation and depression.

In order to control both setting and resetting processes we need to design suitable voltage pulses and to vary the delay between “pre-synaptic” and “post-synaptic” pulses. We chose two different combinations of pulse shape to test the STDP properties of our devices, with programming pulses being the sum of distinct pulses with variable inter-pulse delays. Figure 3 illustrates the concept of changing device conductance by tailoring pulse shape. In the first case, we used square and triangle pulses as the pre- and post-synaptic spikes, respectively (both 1.5 V, Figure 3A). To ensure that the transition point between setting and resetting is at zero the triangle pulse had different rise and fall times (Figure 3A). The leading edge of the triangle pulse was set to be equal to the duration of the square pulse. When the applied pulse, or combination of pulses, is below a threshold voltage, V_th_, there is no change to the device conductance. However, for pulses above V_th_, the device conductance will change dependent on the sequence of voltages applied to the device. In the case of a slowly rising pulse, the device will SET into a low resistance state once the threshold is reached. Rapidly reducing the voltage has little effect, and the device remains in the LRS. However, if the pulse has a sharp leading edge and a slow trailing edge, the integral of current flowing through the conductive filament can become high enough to generate sufficient Joule heating to rupture the filament. The device therefore enters the HRS. In Figure 4 we present an example of 12-cycle set of combined pulses for a non-identical pulse configuration. Each subsequent pulse has the delay between pre- and post-synaptic pulses reduced by 90 μs, and hence the resultant pulse shape changes from square followed by triangle to triangle followed by square. At intermediate delays we can see the device under test initially setting to a LRS and then resetting to a HRS. Device resistance is thus dependent on the time difference between the two pulses. As was predicted, a rising edge above V_th_ lowers the resistance of the device under test, while a gradually descending voltage edge above V_th_ increases it.

**Figure 5:**
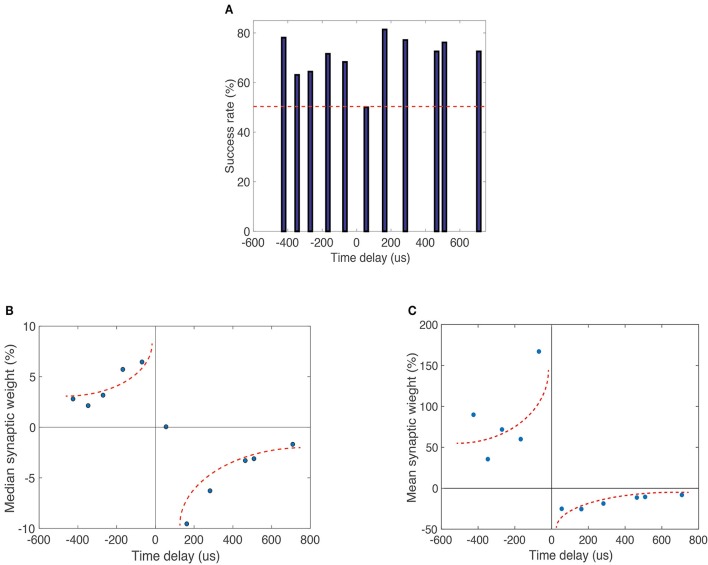
**(A)** Plotted percentage occurrence of expected operation (decrease or increase in conductance for negative and positive time delays, respectively) for 306 independent pulse sets. The occurrence rate is above 50% for most time difference configurations, indicating that expected operations are statistically favorable. **(B)** Median percent change in synaptic weight (conductance) vs. time delay between pre- and post-synaptic spikes for 306 independent identical pulse sets with 11 cycles in each set. **(C)** Mean percent change in synaptic weight (conductance) vs. time delay between pre- and post-synaptic spikes. Data were limited to events that were classified as successes in the statistical analysis shown in **(A)**. Multiple memristive devices were used in this experiment. There is a distinct shape resemblance to biological STDP. Dotted lines are guides for the eye.

In order to generate an STDP-like response with nominally identical pre- and post-synaptic pulses we used two square voltage pulses and modified the post-synaptic pulse with a capacitor; this is a scheme that may easily be implemented in a real system. Our previous work has demonstrated how voltage spikes can be generated by individual RRAM elements, and it would be straightforward to combine these with appropriate capacitive elements. The result is a modified square pulse that looks more like a triangular pulse. This allows us to exploit the previously-shown device response to different leading and trailing pulse edge slopes. To achieve the required gradient we emulated the response of a 3 μF capacitor, but it should be noted that much smaller capacitance values will be sufficient for devices operating in the nanosecond regime rather than the microsecond regime studied here for demonstration. The square pulse peak voltage remained the same at 1.5 V, and the pulse width was 450 μs. The shift that was previously required for a non-identical configuration was no longer needed due to the modification of the post-synaptic pulse. Figure 4B shows an example of a sequence of 11 pulses. The DUT shows similar behavior to the previous setup, and we are able to demonstrate that this pulse setup mimics biological STDP (Figure 5).

### Statistical analysis

We conducted a statistical analysis of the second pulse configuration (square pulse and modified square pulse). The results in Figure 5 are based on 306 pulse cycles identical to those represented in Figure 4. Before applying the pulse trains the devices were electroformed to a preconfigured resistance of ~2 kΩ. The pre-configuration routine is necessary to force a conductive filament into a metastable state whose conductance can be either increased or decreased by the appropriate voltage pulse combination. If the filament were in a very high resistance state, for example, further increases in resistance would not be possible. To investigate the statistical significance of timing-dependent changes in conductance, we determined the success rate of expected occurrences (Figure 5A). Here we measured for the same 306 pulse cycles the probability of a negative difference in pre- to post-synaptic pulse timing resulting in a positive change in synaptic weight (conductance), and vice-versa for positive differences in spike timing. We find that nearly all instances had a success rate >50%, indicating the statistical favorability of STDP-like behavior. The median of synaptic weight change (i.e., change in device conductance) is plotted against the relative time delay between pre- and post-synaptic pulses shows a striking similarity to biological STDP (Figure 5B). There is a transition at small positive delays that mimics biological behavior. Figure 5C shows the mean percent change in synaptic weight (conductance) versus time delay between pre- and post-synaptic spikes. In this case, we selected those instances of successful change in synaptic weight and constructed an STDP response curve. We have thus a proof-of-principle demonstration that not only it is possible to achieve STDP behavior, but also it is a statistically favorable process. We note at this point that the unsuccessful STDP events in our non-optimized devices are primarily related to the variability in set and reset voltages. This stochasticity may be greatly reduced through material optimization and device design, as we have recently demonstrated in RRAM devices for non-volatile memory applications. We are therefore confident that further work will improve substantially the success rates demonstrated here.

## Conclusion

We have shown that it is possible to achieve an STDP response from unipolar SiO_x_ RRAM devices. Furthermore, we have demonstrated that STDP behavior is a statistically favorable occurrence, even in non-optimized devices. This extends and adds to our previous work showing spiking, integration and thresholding in unipolar SiO_x_ RRAM devices (Yu et al., 2011), and allows us to speculate that such devices can be used in hardware spiking neural networks to mimic a broad range of rich neuronal behavior, greatly simplifying the design of hardware neuromorphic systems. Further work is certainly required to establish design rules around neuromorphic systems based on RRAM devices, and the work we report is very much proof-of-concept at this stage. In particular, work is required to determine the flexibility of RRAM devices in terms of control they offer over metrics such as spiking frequency, power dissipation, conductance, and so on. We know from previous work that the electrical response of RRAM devices can be tailored through appropriate programming—particularly by the initial electroforming step—but work is needed to explore the limits of this approach. Further work is also needed to optimize both material and device structure to increase reliability. Nevertheless, combined with excellent CMOS compatibility, silicon oxide unipolar memristive devices are an interesting prospect for future neuromorphic systems.

## Author contributions

KZ: Performed the STDP experiments, designed the study, interpreted the data, and wrote the first draft of the manuscript; AM: Assisted in designing the study, interpreted the data, and assisted with drafting the manuscript; LM and MB: Assisted with experimental design and measurement; SH: Assisted with experiments; AK: Oversaw the work, co-designed the study, and assisted with interpreting the results; All authors contributed to the manuscript.

### Conflict of interest statement

The authors declare that the research was conducted in the absence of any commercial or financial relationships that could be construed as a potential conflict of interest.
